# Early Clinical Performance of Two Powder-Liquid Restoratives in Class-I/II Cavities

**DOI:** 10.3290/j.jad.c_2307

**Published:** 2025-10-17

**Authors:** Line Etiennot, Marcio Vivan Cardoso, Aline Degroote, Bart Van Meerbeek, Marleen Peumans

**Affiliations:** a Line Etiennot PhD Student, KU Leuven, Department of Oral Health Sciences, BIOMAT & UZ Leuven, Dentistry; Kapucijnenvoer 7, Blok a – box 7001, BE-3000 Leuven, Belgium. Evaluation of the restorations, data curation, analysis of 6-month results, and wrote the manuscript.; b Marcio Vivan Cardoso Assistant Professor, KU Leuven, Department of Oral Health Sciences, BIOMAT & UZ Leuven, Dentistry; Kapucijnenvoer 7, Blok a – box 7001, BE-3000 Leuven, Belgium. Coordinating investigator, patient selection, placement of the restorations, and proofread the manuscript.; c Aline Degroote Master in Specialized Oral Health, Restorative Dentistry, KU Leuven, Department of Oral Health Sciences, BIOMAT & UZ Leuven, Dentistry. Kapucijnenvoer 7, Blok a – box 7001, BE-3000 Leuven, Belgium. Patient selection, placement of the restorations, and organizing the 6-month recall.; d Bart Van Meerbeek Full Professor, KU Leuven, Department of Oral Health Sciences, BIOMAT & UZ Leuven, Dentistry; Kapucijnenvoer 7, Blok a – box 7001, BE-3000 Leuven, Belgium. Coordinating investigator, evaluation of the restorations, proofread the manuscript, and contributed to the discussion.; e Marleen Peumans Professor, KU Leuven, Department of Oral Health Sciences, BIOMAT & UZ Leuven, Dentistry; Kapucijnenvoer 7, Blok a – box 7001, BE-3000 Leuven, Belgium. Principal investigator, placement of the restorations, data curation and analysis, proofread the manuscript, and contributed to the discussion.

**Keywords:** Cention Forte, Equia Forte HT, clinical trial, bioactive, alkasite, glass hybrid, bulk fill

## Abstract

**Purpose:**

This randomized clinical trial (RCT) aims to evaluate the clinical performance of the two powder-liquid restoratives Cention Forte (‘CF’; Ivoclar Vivadent AG, Schaan, Liechtenstein) and Equia Forte HT (“EF”; GC, Tokyo, Japan) in class-I/II restorations. Here, the early 6-month clinical performance is reported.

**Materials and Methods:**

Fifty-nine patients were included based on specific exclusion/inclusion criteria. Each patient had two teeth with similar cavities restored using either CF or EF, which were randomly assigned using the Castor EDC system. Baseline (BL) evaluation occurred at 2 weeks followed by a 6-month recall, both conducted by two independent examiners using FDI criteria.

**Results:**

The 6-month recall rate was 96.6%. All restorations were rated as clinically acceptable, except for 1 EF restoration showing a severe but repairable marginal defect. Similar clinical performance was recorded for both restorative materials regarding surface staining, margin discolouration, anatomic form, approximal contact, postoperative hypersensitivity, and tooth integrity. Slight but still clinically acceptable marginal deterioration was observed for both restoratives. Regarding color match, most EF restorations (94.7%) showed a clinically acceptable deviation in color match (opaquer; FDI score 2 and 3), compared to 57.9% CF restorations having an excellent color match rated as FDI score 1. The percentage of EF restorations with a surface luster comparable to that of enamel (FDI score 1) decreased from 50.8% at BL to 14% at 6 months, mainly due to wearing off of the resin-based coating. CF restorations showed more frequently a slightly dull surface (FDI Score 2 for BL: 81.4%; 6 months: 82.5%).

**Conclusion:**

Both powder-liquid restoratives revealed a similarly favorable early clinical performance after 6 months of clinical service.

Dental amalgam has a long clinical history as a reliable, durable, low technique-sensitive, and cost-effective restorative material.^4^ Despite these benefits, it also presents drawbacks, such as poor esthetics, the need for invasive (retentive) tooth preparation, and the inclusion of mercury in the metal alloy, the latter having raised significant public concern.^4,31,46^ In August 2017, the Minamata Convention on Mercury came into effect, calling for the gradual reduction of mercury use, including dental amalgam.^4,46^ More recently, the European Union (EU) announced a complete ban on the use of dental amalgam, which took effect on January 1, 2025.^7^ Consequently, amalgam has largely been replaced by tooth-colored dental resin-based composites (RBCs).^40,46^ Their excellent esthetic outcomes, good long-term clinical performance, and the potential to promote minimally invasive cavity preparation make them a preferred choice for dental restorations.^30,40,45^ However, RBCs also show drawbacks compared to amalgam, as they are highly technique-sensitive and have been associated with a higher incidence of secondary caries.^31,46^ As a result, other materials have been introduced, aiming to combine the benefits of both RBCs (tooth-colored) and amalgam (low technique sensitivity).

One such material is Cention Forte (CF; Ivoclar Vivadent AG, Schaan, Liechtenstein), which is promoted as a new alternative to amalgam and is indicated for use in applications comparable to those of RBCs (information manufacturer).^25^ This novel tooth-colored and claimed bioactive powder-liquid filling material is primarily self-curing, with an optional light-curing feature, allowing it to be applied in bulk. Cention N (‘CN’; Ivoclar Vivadent AG) presents a quite similar composition but differs from CF in two key aspects. First, CF is supplied in capsules, ensuring consistent mixing, whereas CN, delivered in powder-liquid format, requires manual mixing, which may introduce variability. Second, CF requires the previous application of Cention Primer (Ivoclar Vivadent AG) on the cavity walls, while CN can be used without or with a traditional universal adhesive.^22,23^ If no adhesive is used during CN placement, a retentive cavity must be prepared to ensure optimal restoration retention. Both CN and CF are claimed to be bioactive thanks to their ion-releaseing composition, which closely resembles that of RBCs, and their incorporation of Ivoclar’s “alkasite” filler technology.^12^ Consequently, they are both referred to as “alkasite” restorative materials.^21^ Interestingly, this alkasite filler is chemically and structurally very similar to fluorine-containing bioactive glass, which is also used in some glass ionomers (GIs).^21^ Adding fluorine to the glasses of GIs or alkasite materials provides several benefits. First, fluorine lowers differences in the refractive index, enhancing the translucency of the restorative material and allowing it to blend more harmoniously with natural tooth structure.^10,21^ Second, it reduces the melting temperature, improving manufacturing efficiency. Lastly, it enables the release of fluoride and calcium ions while also increasing the pH in the oral environment, which is beneficial for inhibiting the growth of cariogenic bacteria.^21^ The incorporation of bioactive glasses offers the advantage of controlled degradation at a neutral pH, primarily through an ion-exchange mechanism where fluoride ions replace hydroxyl ions from water dissociation in the surrounding environment. This contrasts with the traditionally used fluoro-alumino-silicate fillers in GIs, which require mixing with an acidic solution, primarily polyacrylic acid, to initiate the setting reaction and enable the release of fluoride or other ions, depending on the material composition.^12,21^

Overall, the existing *in-vitro* studies on CF, though limited due to the novelty of the material, show promising results in terms of ion-releasing properties, physico-mechanical characteristics, and biocompatibility.^1,37,38,47^ Additionally, only one *in-vivo *study by Gözetici-Çil et al (2025) reported favourable outcomes after 1 year, noting a statistically significant difference only in colour match (FDI 3a), surface lustre (FDI 1), and surface staining (FDI 2a) compared to the baseline.^16^ In contrast, a small number of *in-vivo *studies have evaluated CN over a one-to-two-year period, reporting satisfactory clinical outcomes.^2,3,5,11,27,35,41^

The second material evaluated in this study is Equia Forte HT (“EF”; GC, Tokyo, Japan), a bulk-fill so-called glass hybrid restorative material introduced in 2019 as a successor to Equia Forte and Equia (GC).^13^ From a broader perspective, EF can be classified as a conventional GI, since it sets through an acid-base reaction, allowing for complete self-curing without the need for additional light-curing.^12,13^ In addition to its fluoride-releasing properties, GIs, like EF, also exhibit self-adhesive properties to dentin. However, a notable limitation is their sensitivity to water and saliva during the early setting stage (<1 h).^21^ Therefore, the manufacturer recommends applying Equia Forte Coat (GC) after placing EF, which requires light curing and provides a protective resin-based layer on the restoration surface.^14,15^ According to the manufacturer, EF is indicated for long-term restorations, including class-I, class-II, and class-V cavities.^13^ Literature has confirmed a good long-term clinical performance of EF or one of its predecessors in class-I and -II restorations, with success reported up to 10 years.^8,17,29,44^ Nonetheless, GIs have been associated with a higher annual failure rate compared to RBCs, which translates into reduced longevity.^8,18^

The aim of this study was to evaluate the clinical performance of CF and EF in class-I and -II cavities in permanent teeth, with the early 6-month performance being reported here. The null hypothesis was that there would be no difference in the clinical success rate of CF and EF in class-I and -II restorations after 6 months.

## MATERIALS AND METHODS

The restorative materials CF and EF were investigated in a prospective, randomized, split-mouth, double-blinded clinical trial. The composition of both materials is detailed in Table 1. This clinical trial was approved by the Commission for Medical Ethics (University Hospitals Leuven, project B3222022000916) and registered in the clinical registry ClinicalTrials.gov (NCT05748327). Patients seeking treatment at the university dental school (UZ/KU Leuven) were recruited for the study based on the inclusion and exclusion criteria outlined in Table 2. Enrollment followed a consecutive approach, with patients included in the order they attended the screening session, resulting in a convenience sample.

**Table 1:** Composition and application procedure of materials used in this study

Material	Batch number (reference)	Composition^1^	Application procedure
**Cention Primer** (Ivoclar Vivadent AG)	Z03RZS (740832 WW)	HEMA, BisGMA, 10-MDP, ethanol, water, D3MA, methacrylate-modified polyacrylic acid, silicon dioxide, camphoroquinone.	Dispense a drop of Cention Primer into a dish and shield it from light. Dip the corresponding single-use coated Cention Primer applicator into the primer and gently stir for approximately 5 s. Scrub onto the cavity surfaces, starting with the enamel, for at least 10 s. Gently air-dry; do not light cure.
**Cention Forte** A2 [CF] (Ivoclar Vivadent AG)	UDMA, calcium fluoro-silicate glass, aromatic- aliphatic UDMA, copolymer, barium-aluminium silicate glass, calcium-barium-aluminium fluoro-silicate glass, DCP, PEG-DMA, barium glass, and ytterbium trifluoride.	Activate the capsule by fully pressing the plunger on a flat surface. Mix it for the recommended duration, based on the room temperature (20–22°C: 17 s; 22–26°C: 15 s; 26–28°C: 13 s) with the capsule mixer (Silamat S6, Ivoclar Vivadent AG). Once mixed, the capsule is placed into the applicator (Capsule Applicator, Ivoclar Vivadent AG), and the material is evenly extruded into the cavity while carefully packing and condensing it. The working time from the start of mixing is 2 min. Light-cure for 15 s, 1,200 mW/cm^2^ using an LED polymerization light (Bluephase G4, Ivoclar Vivadent AG).
**Cavity Conditioner** (GC)		20% polyacrylic acid, 3% aluminum chloride, hexahydrate and distilled water.	Apply to the cavity surfaces for 15 s. Thoroughly rinse with water and gently air-dry.
**Equia Forte HT** [EF] (GC)	A2: 220322A (901574) A3: 220119A (901575) A3.5: 220506A (901587)	Powder: 95% strontium-fluoro-alumino-silicate glass, 5% polyacrylic acid. Liquid: 40% aqueous polyacrylic acid powder.	Shake or tap the capsule against a hard surface to loosen the powder before use. Mix the capsule for 10 s (4,000 rpm; Silamat S6, Ivoclar Vivadent AG). Insert the capsule into the GC capsule applier. Extrude the material evenly into the cavity. Working time is 1 min 30 s from the start of mixing at 23°C. During the first 2 min 30 s after mixing, extra care should be taken to prevent moisture contamination or drying out. The chemical hardening reaction can be fastened by light-curing for 40 s (heat production).
**Equia Forte Coat** (GC)	40–50% methylmethacrylate, 10–15% colloidal silica, 0.09% camphoroquinone, 30–40% urethane methacrylate, 1–5% phosphoric ester monomer.	Apply on the restoration surface using a disposable micro-tip applicator. Immediately light-cure for 20 s.
^1^ As released by the respective manufacturer; BisGMA: bisphenol A-glycidyl methacrylate; DCP: dicalcium phosphate; D3MA: proprietary ingredient; HEMA: 2-hydroxyethyl methacrylate; PEG: polyethyleneglycol; PEG-400-DMA: polyethylene glycol 400 dimethacrylate; UDMA: urethane dimethacrylate; 10-MDP: 10-methacryloyloxydecyl dihydrogen phosphate.

**Table 2:** Inclusion and exclusion criteria

Inclusion criteria	Exclusion criteria
• Patient age between 18 and 65 years old. • Class-I and -II cavities (margin 1–1.5 mm from the cusp tips) to treat primary caries and replace existing defective amalgam or composite restorations in premolars and molars. • Vital teeth. • Two restorations per patient: both cavities should have comparable sizes and dimensions. • Presence of neighboring tooth and antagonist (molars without neighboring tooth at the distal side can also be included). • Low to moderate caries rate; normal periodontal status with good home care.	• Hospitalized and medically compromised patients (medical history may not compromise the outcome of the results). • Pulp exposure or signs of pulpal infection. • No signs of pulpitis or hypersensitivity (Visual Analog Scale (VAS <3). • History of allergy to glass ionomer, acrylate/methacrylate monomers. • Pregnancy. • Chronic disease with oral manifestations or primary oral pathology. • Bad oral hygiene. • High caries rate or periodontal problems. • Absence of antagonists.


In total, 59 patients were selected, aged between 19 and 65 years (mean age: 32.5 years). They were informed about the study’s nature and objectives. Written informed consent was obtained from all participants before initiating treatment. Each patient received both a CF and an EF restoration to allow for a direct comparison of the two restorative materials in one mouth/patient. All 118 restorations were placed to treat dental caries or to replace clinically unacceptable class-I and -II restorations. As specified in the inclusion criteria, the restorations placed in each patient were similar in size and depth. The decision to place either a CF or EF restoration was made through simple randomization using the digital platform Castor EDC (Electronic Data Capture, Amsterdam, the Netherlands) prior to preparing the cavity of the first tooth. This approach aimed to avoid selection bias created by possible operators’ subjective criteria, such as size and/or depth of the cavities.

All restorations were placed between November 2022 and January 2024 by three experienced and specially instructed dentists, trained in restorative procedures. The same operator placed two restorations per patient.

### Restorative Procedure

When necessary, the teeth to be restored were anesthetized with 1.8 ml of 2% lidocaine with 1:80,000 epinephrine (Lignospan 2%, Septodont, St.-Maur, France). In case of a contraindication, Scandonest (30 mg Mepivacainhydrochlorid, Septodont) was used. Isolation was achieved with OptraGate (Ivoclar Vivadent AG), cotton rolls, a saliva ejector, and a suction device (no rubber dam). Cavity preparation was carried out according to tooth-preserving principles. Using CF, the enamel margins were slightly beveled with finishing diamond burs (25–40 μm), as recommended by the manufacturer. In contrast, beveling of the margins was avoided using EF, following common cavity-preparation principles for glass ionomer restorations. In a second step, the cavity was rinsed with water and gently dried with water- and oil-free air. If the cavity included proximal areas, a matrix band (Palodent Plus, Dentsply Sirona, Konstanz, Germany) was placed and secured with a wedge and a Palodent V3 ring (Dentsply Sirona). If the carious lesion was close to the pulp, an indirect pulp-capping procedure was performed before applying the adhesive. Calcium hydroxide (Life, Kerr, Orange, CA, USA) was then used for this purpose, and subsequently covered with pressure-resistant resin-modified glass ionomer lining cement (Fuji Lining LC, GC).

Using CF, Cention Primer (Ivoclar Vivadent AG) was first applied, followed by the placement of CF into the cavity. The application procedure is detailed in Table 1. After shaping and contouring, the restoration was polymerized for 15 s using an LED light-curing unit (Bluephase G4, Ivoclar Vivadent AG) with a light-output of 1200 mW/cm^2^, as measured using MARC Resin Calibrator (Bluelight Analytics, Halifax, Nova Scotia, Canada). Next, excess material was removed using microfine-grit (40 μm) diamond burs or a rubber polishing point (Brownie FG, Shofu, Kyoto, Japan) with water cooling. Proximal excess was removed with flexible finishing discs (Soflex Pop-on discs, 3M Oral Care, Seefeld, Germany) or metal finishing strips (GC New metal strips, GC). Occlusion and articulation were checked, and any necessary adjustments were made using the same instruments as described above. Finally, the restoration was dry-polished at low speed with rubber polishing points (Identoflex yellow rubber point, Kerr).

Using EF, the application procedure followed the manufacturer’s instructions as described in Table 1. First, Cavity Conditioner (GC) was applied to the prepared tooth surface, followed by the placement of EF. The finishing process after setting was carried out in the same way as described for CF. In the final step, Equia Forte Coat (GC) was applied as described in Table 1.

### Evaluation of the Restorations

Two examiners, who were not involved in the restoration placements, evaluated the restorations at baseline (BL: 2 weeks after placement) and 6 months. They were blinded to group assignment. Additionally, patients were also blinded, ensuring a double-blind randomized clinical trial design. The clinicians who placed the restorations were not blinded, as blinding was not feasible due to the different placement procedures required for both materials. Corrections of marginal adaptation and occlusion were still performed just before BL evaluation, when necessary. Clinical photographs were taken of the initial situation, the cavity preparation, the baseline, and 6-month follow-up (Nikon D7200 with Nikon AF-S DX Micro 85 mm lens, Nikon, Tokyo, Japan).

The restorations were evaluated according to the FDI criteria as described by Hickel et al (2007, 2010) and are detailed in Table 3.^19,20^ The restorations were evaluated in terms of esthetic (surface luster and staining, marginal discolouration, color match and translucency, and esthetic anatomic form), functional (fracture of material and retention, marginal adaptation and approximal contact point), and biological properties (patient’s view, postoperative sensitivity, recurrence of caries, tooth integrity, and adjacent mucosa). Any discrepancy in evaluation between the two evaluators was immediately resolved at the chairside.

**Table 3:** FDI evaluation criteria and methodology

Evaluation criterion	Evaluation method
ESTHETIC PROPERTIES
Surface luster (**FDI 1**)	Visually (after air-drying the tooth); the operator light should be switched off, and the evaluation should be carried out at a distance of 60–100 cm. The quality of surface lustre and roughness can only be adequately evaluated if the restored tooth has been thoroughly cleaned and dried.
Surface staining (**FDI 2a**)	Visually (after air-drying the tooth).
Marginal discoloration (**FDI 2b**)	Visually (after air-drying the tooth).
Color match (**FDI 3a**)	Visually (after air-drying the tooth); the operator light should be switched off, and a distance of 60–100 cm is recommended for proper evaluation of colour match.
Translucency (**FDI 3b**)	Visually (after air-drying the tooth); the operator light should be switched off, and a distance of 60–100 cm is recommended for proper evaluation of colour match.
Esthetic anatomic form (**FDI 4**)	Visually (after air-drying the tooth); the operator light should be switched off, and the evaluation should be carried out at a distance of 60–100 cm.
FUNCTIONAL PROPERTIES
Fracture of material and retention (FDI 5)	Visually (after air-drying the tooth) and tactilely using a sharp probe.
Marginal adaptation (FDI 6)	Visually (after air-drying the tooth) and tactilely using a sharp probe.
Approximal contact point (FDI 8)	The proximal contact points can be checked by passing waxed dental floss through the interdental space. A proximal contact point has physiological strength when dental floss or a 25-µm metal blade can pass through it and is evaluated for a certain degree of resistance, resulting in a “snap” effect. Metal matrix strips of different thickness of 25 µm, 50 µm, and 100 µm are used to evaluate the strength of the contact point.
BIOLOGICAL PROPERTIES
Patients view (**FDI 10**)	Anamnese (visual analog scale: “VAS”).
Postoperative sensitivity (**FDI 11**)	Tested using a thermal sensitivity test: a cold carbon dioxide ice stick was held to the tooth to evaluate sensitivity. This should always be compared with testing the reaction of adjacent vital teeth.
Recurrence of caries (**FDI 12**)	Visually and tactilely using a probe (after air-drying the tooth).
Tooth integrity (**FDI 13**)	Visually and tactilely using a probe (after air-drying the tooth).
Adjacent mucosa (**FDI 15**)	Visually (after air-drying the tooth).


When the restoration received a score of 1 to 3, based on the FDI criteria, it was classified as a success. When scores of 4 to 5 were recorded, the restoration was considered a failure. Finally, a score between 1 and 4 indicated that the restoration survived. All data were reported through the cloud-based platform Castor EDC (Electronic Data Capture).

### Sample Size and Statistical Considerations

Expecting a 5-year success rate of 85%, 58 patients were required to estimate this percentage for each procedure with a precision of 10% (half-width of the 95% confidence interval). The study is not powered to detect a statistical difference between the two restorative procedures. The proportion and the exact 95% confidence interval were calculated for the observed survival and success rate at 6 months (Clopper–Pearson exact confidence interval for a proportion based on binomial distribution). A descriptive analysis compared on a pair-wise basis the ratings of the different FDI criteria between CF and EF using the Exact McNemar test at a significance level of 5% (P <0.05).

## RESULTS

**Fig 1:**
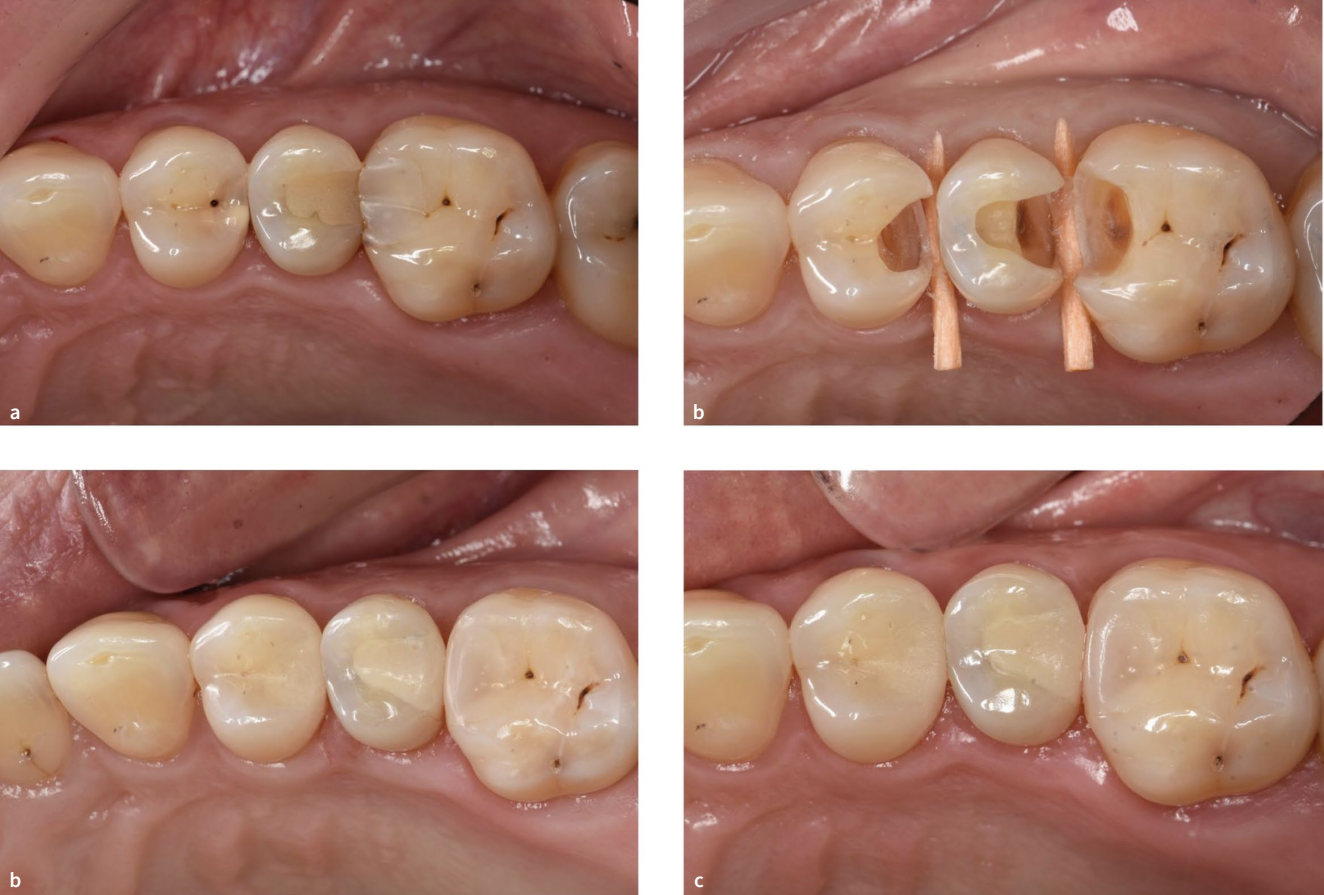
Clinical photographs of a CF (tooth 24) and EF (tooth 25) restoration in one of the patients participating in the RCT. (a) INITIAL clinical situation: tooth 24 presented a carious lesion, while tooth 25 had an old composite restoration with recurrent caries. Tooth 26 was not included in the study and was restored with a conventional composite restoration bonded using an adhesive. (b) Cavity preparation of 24, 25, and 26. (c) BASELINE: tooth 24 was restored with CF, presenting a good colour match and slightly dull surface. Tooth 25, restored with EF, showed a shiny appearance but was more opaque and brighter compared to the natural tooth. (d) 6-MONTH RECALL: the CF restoration maintained a good colour match and slightly dull surface. For the EF restoration, the colour match and translucency remained the same as at BL, while the surface became slightly dull.

In the present study, 59 patients were included at BL. At the 6-month recall, two dropouts were recorded as these patients did not return for the scheduled appointments despite multiple contact attempts, resulting in a recall rate of 96.6%. The preoperative findings, intraoperative findings, and cavity dimensions are detailed in Table 4, Table 5, and Table 6, respectively. The baseline and 6-month results of the clinical investigation are detailed in Table 7. Figure 1 presents clinical pictures of a CF and EF restoration in one of the participants (initial situation, cavity preparation, baseline, and 6-month follow-up).

**Table 4:** Preoperative findings

NUMBER OF TEETH
GENDER
NUMBER OF PATIENTS PER AGE-CATEGORY
INITIAL LESION	Cention Forte	Equia Forte HT
Premolar: 53 (45%)	Molar: 65 (55%)
Female: 31 (52.5%)	Male: 28 (47.5%)
<20 yrs: 1	20–29 yrs: 28	30–39 yrs: 17
40–49 yrs: 8	50–59 yrs: 2	60–65 yrs: 3
Primary caries	45	44
Old composite with caries occurrence	9	9
Old composite without caries occurrence	4	1
Old amalgam with caries occurrence	1	3
Old amalgam without caries occurrence	2	4


**Table 5:** Restoration characteristics

	Cention Forte	Equia Forte HT
Class-I restoration	16 (27.1%)	17 (28.8%)
Class-II restoration	43 (72.9%)	42 (71.2%)
No indirect pulp capping	57 (96.6%)	56 (94.9%)
Indirect pulp capping	2 (3.4%)	3 (5.1%)


**Table 6:** Cavity dimensions and descriptive statistics

Cention Forte	Number	Minimum	Maximum	Average	SD^1^
Equia Forte HT	Number	Minimum	Maximum	Average	SD
Depth of cavity box	46	0	7	3.8	1.1
Occlusal cavity depth	46	0.5	5	3.1	1.1
Buccolingual cavity width	59	1	8	3.8	1.2
Smallest buccolingual cavity width	43	1	5	2.1	0.9
Depth of cavity	43	3	7	3.8	0.9
Occlusal cavity depth	46	1	7	3.1	1.0
Buccolingual cavity width	58	2	7	3.9	1.4
Smallest buccolingual cavity width	44	1	4	1.9	0.8
SD: Standard deviation.

**Table 7:** Results of the baseline and 6-month recall in percentage (n) following the FDI evaluation criteria

ESTHETIC PROPERTIES
	Cention Forte	Equia Forte HT
% (n)	BASELINE	6 MONTHS	BASELINE	6 MONTHS
**Surface lustre (FDI 1)**
Clinically excellent	13.5 (8)	1.7 (1)	50.8 (30)	14 (8)
Clinically good	81.4 (48)	82.5 (47)	44.1 (26)	64.9 (37)
Clinically sufficient	5.1 (3)	15.8 (9)	5.1 (3)	21.1 (12)
Clinically insufficient	0 (0)	0 (0)	0 (0)	0 (0)
Clinically poor	0 (0)	0 (0)	0 (0)	0 (0)
**Surface staining (FDI 2a)**
Clinically excellent	100 (59)	94.7 (54)	100 (59)	89.5 (51)
Clinically good	0 (0)	5.3 (3)	0 (0)	10.5 (6)
Clinically sufficient	0 (0)	0 (0)	0 (0)	0 (0)
Clinically insufficient	0 (0)	0 (0)	0 (0)	0 (0)
Clinically poor	0 (0)	0 (0)	0 (0)	0 (0)
**Marginal discoloration (FDI 2b)**
Clinically excellent	100 (59)	86 (49)	96.6 (57)	89.5 (51)
Clinically good	0 (0)	12,3 (7)	3.4 (2)	10.5 (6)
Clinically sufficient	0 (0)	1.7 (1)	0 (0)	0 (0)
Clinically insufficient	0 (0)	0 (0)	0 (0)	0 (0)
Clinically poor	0 (0)	0 (0)	0 (0)	0 (0)
**Colour match (FDI 3a)**
Clinically excellent	59.3 (35)	57.9 (33)	8.5 (5)	5.3 (3)
Clinically good	40.7 (24)	42.1 (24)	67.8 (40)	75.4 (43)
Clinically sufficient	0 (0)	0 (0)	23.7 (14)	19.3 (11)
Clinically insufficient	0 (0)	0 (0)	0 (0)	0 (0)
Clinically poor	0 (0)	0 (0)	0 (0)	0 (0)
**Color change (FDI 3a)**
Darker	58.6 (17)	72.4 (21)	24.1 (13)	31.5 (17)
Brighter	41.4 (12)	27.6 (8)	75.9 (41)	68.5 (37)
**Translucency (FDI 3b)**
Clinically excellent	54.2 (32)	36.8 (21)	1.7 (1)	3.5 (2)
Clinically good	45.8 (27)	63.2 (36)	83.1 (49)	82.5 (47)
Clinically sufficient	0 (0)	0 (0)	15.3 (9)	14 (8)
Clinically insufficient	0 (0)	0 (0)	0 (0)	0 (0)
Clinically poor	0 (0)	0 (0)	0 (0)	0 (0)
**Translucency change (FDI 3b)**
More opaque	100 (32)	97.4 (37)	98.3 (58)	100 (56)
More translucent	0 (0)	2.6 (1)	1.7 (1)	0 (0)
Esthetic anatomic form (FDI 4)
Clinically excellent	72.9 (43)	86 (49)	67.8 (40)	75.4 (43)
Clinically good	27.1 (16)	14 (8)	32.2 (19)	24.6 (14)
Clinically sufficient	0 (0)	0 (0)	0 (0)	0 (0)
Clinically insufficient	0 (0)	0 (0)	0 (0)	0 (0)
Clinically poor	0 (0)	0 (0)	0 (0)	0 (0)

**Table:** 

FUNCTIONAL PROPERTIES
	Cention Forte	Equia Forte HT
% (n)	BASELINE	6 MONTHS	BASELINE	6 MONTHS
FUNCTIONAL PROPERTIES
	Cention Forte	Equia Forte HT
% (n)	BASELINE	6 MONTHS	BASELINE	6 MONTHS
**Fracture material and retention (FDI 5)**
Clinically excellent	100 (59)	98.2 (56)	96.6 (57)	89.5 (51)
Clinically good	0 (0)	0 (0)	1,7 (1)	1,7 (1)
Clinically sufficient	0 (0)	1.7 (1)	1.7 (1)	7 (4)
Clinically insufficient	0 (0)	0 (0)	0 (0)	1.7 (1)
Clinically poor	0 (0)	0 (0)	0 (0)	0 (0)
**Marginal adaptation (FDI 6)**
Clinically excellent	91.5 (54)	59.6 (34)	84.7 (50)	66.7 (38)
Clinically good	8.5 (5)	40.4 (23)	15.3 (9)	28.1 (16)
Clinically sufficient	0 (0)	0 (0)	0 (0)	3.5 (2)
Clinically insufficient	0 (0)	0 (0)	0 (0)	1.7 (1)
Clinically poor	0 (0)	0 (0)	0 (0)	0 (0)
**Approximal contact point (FDI 8)**
Clinically excellent	100 (75)	98.6 (71)	97.2 (74)	95.9 (70)
Clinically good	0 (0)	1.4 (1)	1.4 (1)	1.4 (1)
Clinically sufficient	0 (0)	0 (0)	1.4 (1)	2.7 (2)
Clinically insufficient	0 (0)	0 (0)	0 (0)	0 (0)
Clinically poor	0 (0)	0 (0)	0 (0)	0 (0)

**Table:** 

BIOLOGICAL PROPERTIES
	Cention Forte	Equia Forte HT
% (n)	BASELINE	6 MONTHS	BASELINE	6 MONTHS
**Patients’ view (FDI 10)**
VAS score 0	81.4 (48)	88.1 (52)	81.4 (48)	88.1 (52)
VAS score 1	15.2 (9)	5.1 (3)	13.5 (8)	5.1 (3)
VAS score 2	3.4 (2)	1.7 (1)	5.1 (3)	1.7 (1)
VAS score 3	0 (0)	1.7 (1)	0 (0)	1.7 (1)
No info	0 (0)	3.4 (2)	0 (0)	3.4 (2)
**Postoperative sensitivity (FDI 11)**
Clinically excellent	86.4 (51)	87.7 (50)	84.7 (50)	93 (53)
Clinically good	11.9 (7)	12.3 (7)	13.6 (8)	5.4 (3)
Clinically sufficient	1.7 (1)	0 (0)	1.7 (1)	1.8 (1)
Clinically insufficient	0 (0)	0 (0)	0 (0)	0 (0)
Clinically poor	0 (0)	0 (0)	0 (0)	0 (0)
**Recurrence of caries (FDI 12)**
Clinically excellent	100 (59)	100 (57)	100 (59)	100 (57)
Clinically good	0 (0)	0 (0)	0 (0)	0 (0)
Clinically sufficient	0 (0)	0 (0)	0 (0)	0 (0)
Clinically insufficient	0 (0)	0 (0)	0 (0)	0 (0)
Clinically poor	0 (0)	0 (0)	0 (0)	0 (0)
**Tooth integrity (FDI 13)**
Clinically excellent	81.3 (48)	86 (49)	89.9 (53)	84.2 (48)
Clinically good	15.3 (9)	10,5 (6)	8.5 (5)	12.3 (7)
Clinically sufficient	3.4 (2)	3.5 (2)	1.7 (1)	3.5 (2)
Clinically insufficient	0 (0)	0 (0)	0 (0)	0 (0)
Clinically poor	0 (0)	0 (0)	0 (0)	0 (0)
**Adjacent mucosa (FDI 15)**
Clinically excellent	94.9 (56)	59.6 (34)	98.3 (58)	59.6 (34)
Clinically good	5.1 (3)	40.4 (23)	1.7 (1)	40.4 (23)
Clinically sufficient	0 (0)	0 (0)	0 (0)	0 (0)
Clinically insufficient	0 (0)	0 (0)	0 (0)	0 (0)
Clinically poor	0 (0)	0 (0)	0 (0)	0 (0)


The inter-evaluator agreement was 88% at BL and 90% at 6 months, with surface luster (FDI 1), color match (FDI 3a), translucency (FDI 3b), and marginal adaptation (FDI 6) requiring the most compromises.

**Fig 2a and b:**
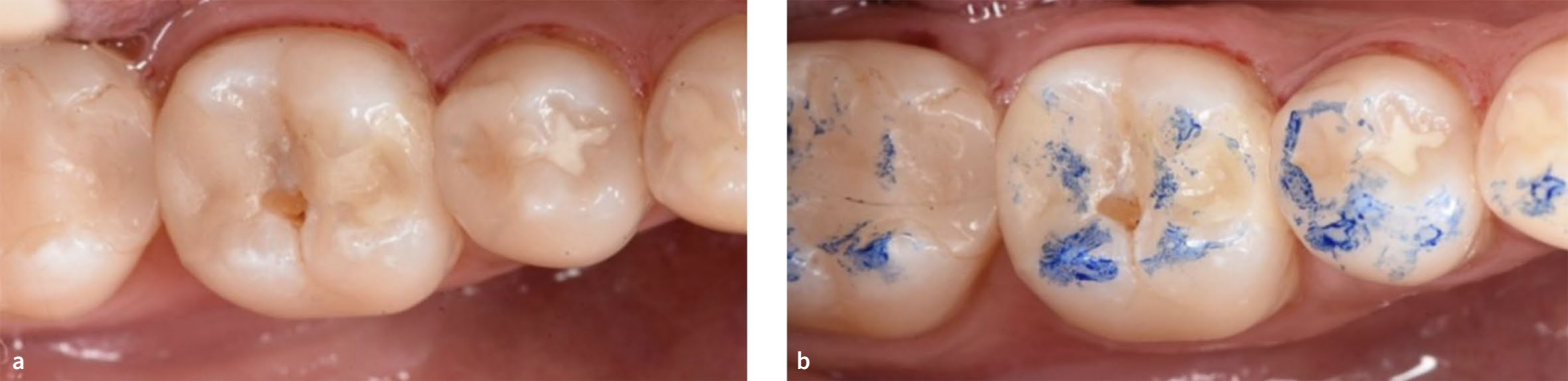
At 6-month recall: (a) the EF restoration (MOD) on tooth 46 showed a severe marginal defect/chip fracture at the occlusal surface and was scored as ‘clinically insufficient’. The defect may have resulted from an underlying air bubble during the initial placement of the restoration. (b) The defect at the occlusal surface was repaired with EF.

At the 6-month recall, both restorative materials generally achieved excellent and comparable scores for the different FDI criteria, similar to BL, with the exception of surface luster (FDI 1), color match (FDI 3a), translucency (FDI 3b), and marginal adaptation (FDI6). All restorations were rated as clinically acceptable, except for one EF restoration showing a chip fracture causing a severe but repairable marginal defect (Fig 2). This resulted in a 100% survival rate for both materials, a 100% (95% CI: 94–100%) success rate for CF, and 98% (95% CI: 91–100%) success rate for EF.

Regarding marginal adaptation (FDI6), slight marginal deterioration was observed for both materials (P >0.05). This was reflected in a decrease in the percentage of restorations with an excellent marginal adaptation (score 1) (CF: Δ% = 31.9, EF: Δ% = 18) and an increase in the percentage of restorations showing a slight clinically acceptable marginal defect (score 2 and 3) (CF: Δ% = 31.9, EF: Δ% = 16.3). These marginal defects presented mainly as irregularities. As mentioned above, all restorations remained clinically acceptable, except for one EF restoration showing a marginal gap at the occlusal surface due to fracture of the restorative material (Fig 2). This restoration was repaired at the 6-month recall with EF.

**Fig 3:**
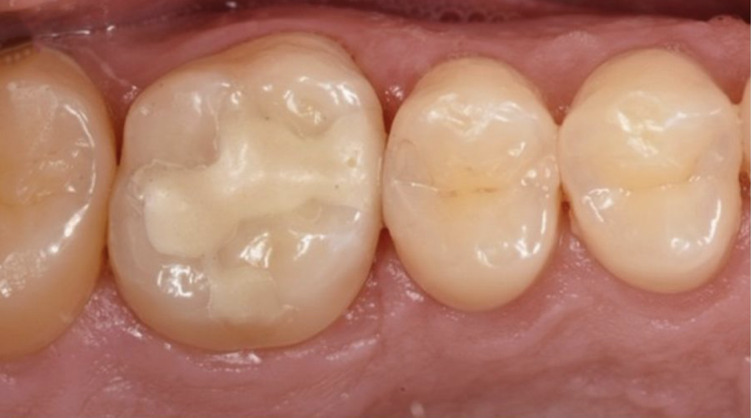

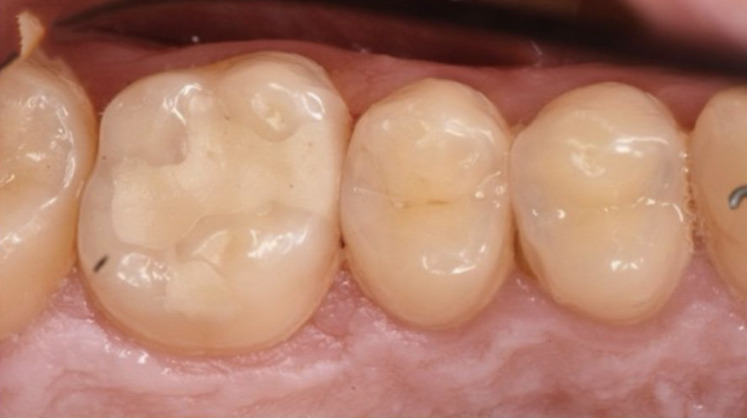

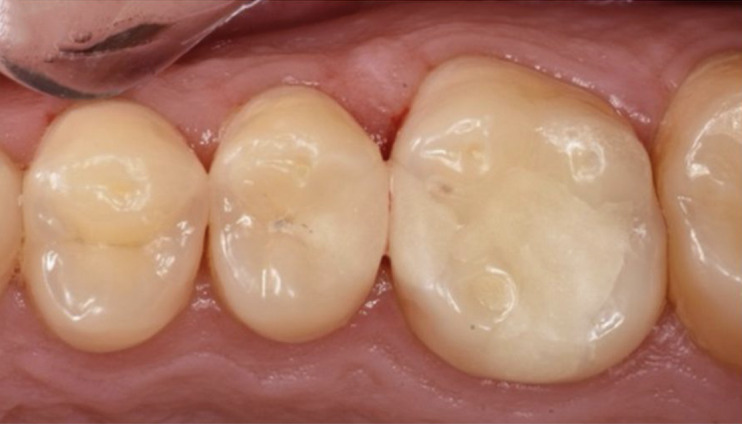

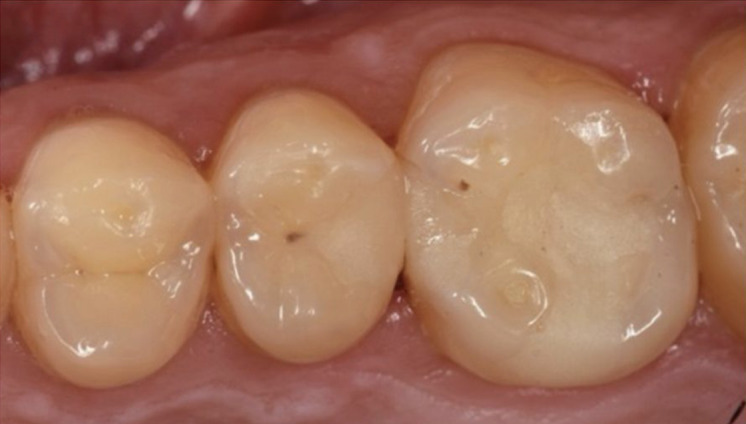
The first maxillary molars (16 and 26) in this patient were restored with restorations of similar size (MOP). (a) Baseline: tooth 16 was restored with EF. The restoration was more opaque and brighter compared to the surrounding natural tooth structure. (b) 6-month recall: the opacity and brightness remained the same; however, the surface of the restoration became slightly dull due to the wearing off of the Equia Forte Coat (GC) resin coating. (c) Baseline: tooth 26 was restored with CF. The restoration showed a slight deviation in colour match (too bright) and translucency (opaquer). The surface was slightly dull. (d) 6-month recall: the abovementioned evaluation criteria were scored in the same way (opaquer, too bright, and slightly dull surface).

At BL, EF restorations demonstrated a superior surface luster (FDI1 score 1) in 50.8% of the cases, compared to 13.5% for CF (Fig 3) (P = 0.02). The surface luster of the EF restorations, however, significantly decreased after 6 months of clinical functioning, shifting from clinically excellent to clinically good or sufficient (score 2 and 3) (CF: Δ% = 11.8 vs EF: Δ% = 36.8). CF restorations also exhibited a decrease in surface luster; however, the decline was less pronounced.

Regarding color match (FDI3a) (P <0.001) and translucency (FDI3b) (P <0.001), CF demonstrated a significantly higher percentage of restorations achieving an excellent color match (score 1) (CF: 57.9% vs EF: 5.3%) and translucency (CF: 36.8% vs EF: 3.5%) at the 6-month recall. In both materials, deviations in translucency were almost always observed as increased opacity (Fig 3).

For the other FDI criteria, like surface staining (FDI 2a), marginal discolouration (FDI 2b), anatomic form (FDI 4), approximal contact (FDI 8), postoperative hypersensitivity (FDI 11), caries recurrence (FDI 12), and tooth integrity (FDI 13), both restorative materials exhibited similar clinical performance (P >0.05).

## DISCUSSION

This study evaluated the early 6-month clinical performance of CF compared to EF in class-I and -II cavities. Only class-I and -II cavities were selected, as this is a requirement for EF, given that larger cavities are contraindicated for its use. Identifying two class-I or -II cavities of similar dimensions within the same patient proved to be a challenging task, which made recruiting an adequate patient population for this study particularly demanding.

Furthermore, given the novelty of CF, which was launched in April 2021, this is the second clinical trial to investigate its performance.^24^ Overall, the results for CF in our study were consistent with those reported in the 1-year *in-vivo* investigation by Gözetici-Çil et al (2025).^16^ In contrast, CN, which was marketed earlier, has been more extensively investigated, with six short-term clinical trials conducted to date in class-I and/or class-II cavities.^2,5,11,27,35,41^ Kataria et al (2023) assessed the performance of CN over a 1-year period in class-I cavities of primary dentition.^27,41^ In contrast, the other studies examined CN in adult permanent dentition, with assessment periods ranging from 6 months to 2 years.^2,5,11,35^ Notably, all studies focused on adult populations except for Sharma et al (2023), who investigated CN in permanent molars of children aged 7 to 13 years.^41^ A systematic review of *in-vitro* studies by Justen et al (2024) reported that CN shared similar properties with conventional RBCs, including load-to-fracture, compressive strength, hardness, and color stability. The review also revealed that CN exhibited superior performance compared to GIs, demonstrating higher compressive, diametral, and flexural strength.^26^ Although the composition of CF and CN is almost similar, the hand-mixing manipulation of CN may compromise its physico-mechanical properties.^34^ As a result, direct comparisons between CF and CN should be approached with caution.^26^ Additionally, in the clinical trials by Oz et al (2023) and Bozkurt et al (2024), no adhesive was used during CN placement.^5,35^ Oz et al (2023) observed a retention loss of 3% at 6 months and 5.1% at 1 year for CN restorations. Similarly, Bozkurt et al (2024) reported four partial retention losses out of the 65 fillings after 2 years. In all other clinical trials evaluating the performance of CN, cavities were selectively etched with phosphoric acid, followed by the application of a universal adhesive, most commonly Tetric N-Bond Universal (Ivoclar Vivadent AG).^2,11,27,41^ Only Albelasy et al (2024) reported a single case of retention loss.^2^ However, the authors attributed this outcome to a void likely introduced during the hand-mixing process, highlighting a potential technique-sensitive aspect of CN preparation. In our study, where Cention Primer (Ivoclar Vivadent AG) was used prior to CF placement, no retention loss was observed for CF after 6 months. Additionally, Samy et al (2025) and Sadeghyar et al (2022) reported a significantly higher shear bond strength of CF to dentin when Cention Primer was applied.^36,39^ This bond strength was notably superior compared to CF applied without primer and other self-adhesive materials, highlighting the primer’s contribution in enhancing material retention and durability.

In the present study, all restorations were clinically successful after 6 months, except for one EF restoration, which exhibited a severe but repairable marginal defect (Fig 2). This resulted in a survival rate of 100% for both restorative materials. A similar clinical performance was observed for both restorative materials across the following FDI criteria: surface staining, marginal discolouration, anatomic form, approximal contact, postoperative hypersensitivity, caries recurrence, and tooth integrity. The satisfactory 6-month clinical performance of both restorative materials in the present study aligns with findings from several other short- to medium-term clinical trials.^2,8,11,27,29,41,43,44^

Furthermore, rubber dam isolation was not used in this study, as relative isolation was recommended by the manufacturer. This highlights the lower technique sensitivity of the CF and EF restorative materials, making them comparable to amalgam in this regard.^14,22^ Sharma et al (2023) placed CN restorations under rubber dam isolation and evaluated their performance after 1 year. However, the results did not appear significantly different from other clinical studies where CN was placed without rubber dam isolation.^38^

Regarding marginal adaptation (FDI6), clinically acceptable marginal deterioration was observed for both restoratives. Using EF, two moderate marginal defects (score 3) and one clinically unacceptable but repairable defect (score 4) were recorded, whereas CF exhibited only slight marginal defects (score 2). Interestingly, our study observed a higher percentage of score 2 for marginal adaptation of both restorative materials after 6 months compared to existing literature.^2,5,8, 16,27,35,41^ Since marginal adaptation is evaluated visually and tactilely using a sharp probe, and considering that baseline marginal adaptation was excellent for 91.5% of CF and 84.7% of EF restorations, whereas the literature almost always reports 100%, this discrepancy could be attributed to the stricter evaluation criteria applied by the two appointed evaluators in our study. Moreover, the higher occurrence of moderate and severe defects for EF compared to CF can be attributed to the superior physico-mechanical properties of CF over those of the GI EF. Only one study evaluated the mechanical properties of CF *in vitro*, yielding seemingly adequate results. However, CF was not compared to a well-established reference.^1^ In contrast, CN has been studied more extensively and was reported to exhibit better physico-mechanical properties than GIs and even amalgam.^26^ Furthermore, it is worth noting that in this study, CF was used in dual-cure mode, with the restoration being light-cured for 15 s after placement. The* in-vitro* study of Negovetic Mandic et al (2024) suggested that light-curing CF restorations significantly improved the physico-mechanical properties compared to self-cure mode.^33^ However, interestingly, the clinical trials of Oz et al (2023), Albelasy et al (2024), and Bozkurt et al (2024) achieved successful clinical performance at 1 and 2 years despite not having light-cured the CN restorations upon placement.^2,5,35^ This suggests that while light-curing may improve material properties in laboratory settings, its impact on long-term clinical outcomes requires further investigation.

Furthermore, the percentage of EF restorations exhibiting a surface luster comparable to that of enamel declined from 50.8% at baseline to 14% at 6 months (Fig 3). This reduction can primarily be attributed to the gradual wear of the resin-based Equia Forte Coat (GC), which is designed to create a smooth, glossy surface while also enhancing physico-mechanical properties and improving marginal adaptation.^6,37,42^ This wearing away of the protective coat is consistent with the literature. The study of Türkün and Kanik (2016) reported its disappearance within 6 months, leading to the loss of its glossy appearance.^8,42,43^ Nevertheless, it is assumed that by the time the coating has worn off, the material has fully matured and become more resistant to water-balance fluctuations while having reached its peak physico-mechanical properties.^6,42^ The meta-analysis of Cribari et al (2023) reported that the resin-based coat provided effective protection against wear by gradually wearing away itself.^8,9^ In contrast, no coating material was indicated for CF. Additionally, CF restorations proved challenging to polish to a high gloss, resulting in a dull surface luster at BL in more than 80% of the restorations. However, changes in surface appearance after six months were minimal. A plausible explanation for this could be the presence of larger filler particles in CN, ranging from 0.1 to 35 µm.^32^ These larger particles result in a more irregular surface. Additionally, during polishing, some filler particles could come loose, creating microscopic voids that further hinder the achievement of a smooth, highly polished surface. Moreover, CF demonstrated superior performance compared to EF in terms of color match (FDI 3a; score 1: CF: 57.9% vs. EF: 5.3%) and translucency (FDI 3b; score 1: CF: 36.8% vs EF: 3.5%). However, the remaining 94.7% of EF restorations were rated as clinically acceptable (scores 2 and 3). The slight color deviations observed in CF restorations (scores 2 and 3: 42.1%) were likely due to the fact that CF is currently only available in shade A2, limiting its ability to match all tooth shades.^24^ In general, GIs have been reported to exhibit poorer esthetics compared with RBCs, primarily due to their reduced translucency.^17,29,42–44^ Furthermore, the color of GIs can change over time, either improving or worsening the match with the surrounding tooth structure.^43,44^ In contrast, the studies by Oz et al (2023) and Kataria et al (2023) both reported an overall good color match for CN in class-II and -I cavities, respectively. Only a slight decrease of 3–5% in color match was reported after 12 months.^27,35^ Additionally, the meta-analysis of *in-vitro* studies by Justen et al (2024) indicated that bioactive composites, including so-called alkasite materials, demonstrated good color stability.^26^ Regarding translucency, most restorations in both materials that did not perfectly match, were rated as more opaque. However, CF restorations with a slight color mismatch were more frequently reported as darker, whereas EF restorations were more often perceived as brighter. Furthermore, it is important to note that in the present study, the color match remained scored as acceptable in all restored teeth (in both CF and EF) after 6 months of clinical service.

Regarding postoperative hypersensitivity (FDI 11), minor sensitivity (score 2 and 3) was reported in 7 CF and 4 EF restorations at the 6-month recall. Notably, EF showed a slight decrease in sensitivity compared to baseline, while CF exhibited consistent results over time. For CN, the clinical trials of Oz et al (2023) and Sharma et al (2023) reported no postoperative sensitivity up to 1 year.^35,41^ However, Hirani et al (2018) observed increased postoperative sensitivity one month after placement in class-I cavities for CN restorations placed without an adhesive. Additionally, in the clinical trial of Albelasy et al (2024), CN, when used with an adhesive in class-I and -II cavities, exhibited an FDI score of 2 for postoperative sensitivity in 50% of the restorations at baseline. Notably, no postoperative sensitivity was reported at the 6-month recall.^2^

Consistent with the literature, no secondary caries was observed in our study. However, a 6-month evaluation period is too short to fully assess the occurrence of secondary caries. A longer evaluation period is needed to better understand the potential cariostatic effects of both CF and EF, particularly considering their fluoride-releasing capabilities, which may take more time to show a significant benefit in preventing caries.^17,38,42^ Currently, studies have reported an adequate release of Ca^2+^ and F- ions out of both materials.^28,38^ Šalinović et al (2023) found that EF showed higher remineralization potential compared to CF. However, this study evaluated microhardness on dentin after just 28 days, which can be considered a relatively short period. Given that CF is a dual-cure restorative material, it is expected that its fluoride release occurs at a slower rate compared to self-cure materials like EF.^38^

In summary, the null hypothesis, which stated there would be no difference in the success rates between CF and EF class-I and -II restorations after 6 months, can be accepted. Additionally, while the clinical study covered a relatively short period of 6 months, it remains one of the first clinical investigations of CF, offering valuable initial insights into its clinical performance. Longer-term (up to 5-year) follow-up of the restorations is planned in order to confirm that both powder-liquid restorative materials can be regarded as amalgam alternatives in class-I and -II load-bearing restorations.

## CONCLUSION

The so-called alkasite powder-liquid restorative Cention Forte (Ivoclar Vivadent AG) and the so-called “glass hybrid” restorative Equia Forte HT (GC) revealed similar early and favorable clinical performance in class-I and -II cavities after 6 months of clinical service.

### Clinical Relevance

After 6 months of clinical functioning, Cention Forte (Ivoclar Vivadent AG) and Equia Forte HT (GC) can be considered as promising tooth-colored low technique-sensitive powder-liquid restorative materials for class-I and -II restorations.

### Acknowledgments

This randomized clinical trial is supported by Ivoclar Vivadent AG (Schaan, Liechtenstein).
